# A Multiple-Center, Retrospective Study of Characteristics and Outcomes of Hospitalized COVID-19 Patients with Cardiovascular Disease in North Iran

**DOI:** 10.4314/ejhs.v33i1.2

**Published:** 2023-01

**Authors:** Abazar Pournajaf, Mehrdad Halaji, Farzin Sadeghi, Mohammad Chehrazi, Mostafa Javanian, Masoumeh Bayani, Mahmoud Sadeghi Haddad Zavareh, Mehran Shokri, Mohsen Mohammadi, Iraj Jafaripour, Roghayeh Pourkia, Mehrdad Saravi, Kamyar Amin, Naghmeh Zieaie Amiri, Mohammad Taghi Hedayati Goudarzi, Farzad Jalali, Yousef Yahyapour

**Affiliations:** 1 Infectious Diseases and Tropical Medicine Research Center, Health Research Institute, Babol University of Medical Sciences, Babol, Iran; 2 Cellular and Molecular Biology Research Center, Health Research Institute, Babol University of Medical Sciences, Babol, Iran; 3 Department of Biostatistics and Epidemiology, School of Public Health, Babol University of Medical Sciences, Babol, Iran; 4 Non-Communicable Pediatric Diseases Research Center, Health Research Institute, Babol University of Medical Sciences, Babol, Iran; 5 Department of Cardiology, School of Medicine, Rouhani Hospital, Babol University of Medical Sciences, Babol, Iran

**Keywords:** COVID-19, cardiac disease, SARS-CoV-2, cardiovascular disease, Iran

## Abstract

**Background:**

In this retrospective study, we investigated the outcomes and demographic characteristics of COVID-19 patients with and without a history of CVD.

**Methods:**

This large retrospective, multicenter study was performed on inpatients with suspected COVID-19 pneumonia who were admitted across four hospitals in Babol, Northern Iran.

Demographic data, clinical data, and cycle threshold value (Ct) results of Real Time PCR were obtained. Then, participants were divided into two groups: (1) cases with CVDs, (2) cases without CVDs.

**Results:**

A total of 11097 suspected COVID-19 cases with a mean ± SD age of 53 ±25.3 (range: 0 to 99) years were involved in the present study. Out of whom 4599 (41.4%) had a positive RT-PCR result. Of those, 1558 (33.9%) had underlying CVD. Patients with CVD had significantly more co-morbidities such as hypertension, kidney disease, and diabetes. Moreover, 187 (12%) and 281 (9.2%) of patients with and without CVD died, respectively. Also, mortality rate was significantly high among the three groups of Ct value in patients with CVD, with the highest mortality in those with Ct between 10 and 20 (Group A = 19.9%).

**Conclusions:**

In summary, our results highlight that CVD is a major risk factor for hospitalization and the severe consequences of COVID-19. Death in CVD group is significantly higher compared to non-CVD. In addition, the results show that age-related diseases can be a serious risk factor for the severe consequences of COVID-19.

## Introduction

Coronavirus disease 2019 (COVID-19) as a pandemic viral disease is a severe acute respiratory syndrome coronavirus 2 (SARS-CoV-2) (1). It was firstly observed in December 2019 in Wuhan, China, rapidly spreading in the world and critically challenging the medical communities and public health. It has had also a negative effect on Iran (2, 3). During influenza outbreaks, it had been reported that several infected patients died because of complications of comorbid cardiovascular disease (CVD) than for secondary bacterial pneumonia (4, 5).

Comorbidities have been observed in a large number of COVID-19 patients, and CVD is the most frequently reported one, which has been observed in about 30–48 percent of patients (6, 7). It seems that those with pre-existing CVD are more vulnerable to developing COVID-19 and experience the disease in a higher severity with worse clinical outcomes. There is often an association between existence of CVD and other comorbidities predisposing patients to severe and more frequent infection forms (8, 9).

Several potential possibilities have been proposed for association between CVD and adverse clinical outcomes in COVID-19 patients. Firstly, the accumulation of CVD risk factors and conditions (for example, diabetes mellitus, smoking, obesity) increases the risk of short-term mortality in hospitalized COVID-19 patients in a progressive manner (10, 11) and can affect pulmonary and infectious reserve, accounting for higher rate of mortality(12). Secondly, CVD and related risk factors, e.g., diabetes, could cause functional immunosuppression leading to predisposition to more severe infectious diseases (13, 14). As reported by a recent research, hospitalized COVID-19 subjects with concomitant cardiac disease show a greatly poor prognosis in comparison with patients without cardiac disease (15, 16).

As recent studies have shown, the cycle threshold value (Ct) results are directly affected by a variety of parameters such as sample type, sampling time, assay design, and report interpretation, and should thus be evaluated with caution. Furthermore, viral load in COVID-19 may be associated with infectivity, disease phenotype, morbidity, and death (17, 18).

Therefore, although it seems that those with a background of cardiac disease are more likely to be infected and experience a more severe COVID-19 course (19), little evidence is present regarding the outcomes and characteristics of the hospitalization of such patients in Iran. In the current work, the outcomes and demographic characteristics of COVID-19 patients with CVD are described and they are compared with COVID-19 patients without a history of CVD hospitalized at the same hospital for the same period.

## Methods

**Patients and study design**: This large retrospective, multicenter study included 11097 inpatients with suspected COVID-19 pneumonia who were admitted across four hospitals in Babol, Northern Iran (Ayatollah Rohani, Shahid Beheshti, Shahid Yahyanejad and Amirkola Children Hospital) affiliated to Babol University of Medical Sciences (MUBABOL) between 7 March 2020 and 20 December 2021(20).

This study was approved by the ethics committee of Babol University of Medical Sciences, Babol, Iran with ethics code IR.MUBABOL.REC.1400.012.

**Data collection**: A COVID-19 patient was included in the dataset if tested positive for COVID-19 infection using a real-time reverse-transcriptase polymerase chain-reaction (RT-PCR) test. Demographic data, clinical data (comorbidities, complications, and outcomes data), were obtained from electronic medical records and cycle threshold value (Ct) results were collected from Molecular Virology Lab by our investigators. The records of patients with considerable missing values, duplicates cases and unclear RT-PCR results and information were excluded. Finally, participants were divided into two groups: (1) cases with CVDs, (2) cases without CVDs. The diagnosis of any CVDs was confirmed via patients' previous documents.

**Laboratory confirmation**: Immediately after admission, laboratory diagnostic of suspected COVID-19 cases was performed using RT-PCR assay of oropharyngeal and nasopharyngeal swab specimens. Viral RNA was extracted using the Ribospin vRD plus Kit (GeneAll, Seoul, South Korea) according to the manufacturer's protocol. Then, RT-PCR was performed to detect the presence of SARS-CoV-2 using ABI 7300 Real Time PCR System. The relative viral loads of SARS-CoV-2 samples were estimated with Ct based RT-PCR results. Based on diagnostic Ct values, Patients were divided into three groups: (group A): Ct 10–20, (Group B): Ct 21–30, and (Group C): Ct 31–40, (21, 22).

**Statistical analysis**: SPSS software, version 16 (SPSS Inc., Chicago, IL, USA) was used to analyze the data. Continuous variables with a normal distribution were expressed as mean ± standard deviation (SD), alternatively, the median and interquartile range was effectively used for non-normal distribution measures. Moreover, Chi-square and Fisher's exact tests were used to perform intergroup and categorical comparisons as appropriate. P-values of < 0.05 were considered statistically significant.

## Results

A total of 11097 suspected COVID-19 cases (49.1% female & 50.9% male) with a mean ± SD age of 53 ±25.3 (range: 0 to 99) years were involved in the present study. Out whom 4599 (41.4%) had a positive RT-PCR result. Of those, 1558 (33.9%) had underlying CVD. The mean age in CVD and non- CVD groups was 65.9±13.6 and 52±19.5 years, respectively. The male subjects were 45.2% in CVD and 51.2% in the non- CVD groups. There was significant difference between the age and of the two groups (P-value ≤ 0.05).

The details of patients with underlying CVD and non- CVD are presented in Table 1. As shown in Figure 1, most of patients with CVD were 45–64 years old (38.9%; 606/1558) while the lowest patients belonged to 1≥ years (0.1%; 2/1558). Patients with CVD had significantly more co-morbidities such as hypertension, kidney disease (KD), and diabetes (DM). Moreover, 34.6% of the patients had only one comorbidity, while 65.4% of patients had more than one comorbidity. Moreover, 187 (12%) and 281 (9.2%) of hospitalized patients with and without CVD died, respectively. Statistical analysis showed that the presence of at least one comorbidity was significantly different in the two groups (*p*≤0.01) as shown in Table 1.

**Table 1:** Demographic and comorbidities information of COVID-19 patients with and without CVD

Variable	Total (n=4599) Number (%)	With CVD (n=1558) Number (%)	Without CVD (n=3041) Number (%)	*p*-value
**Age (Mean ±SD)**		65.9±13.6	52±19.5	<0.01
**Sex**				
Male	2260 (49.1)	704 (45.2)	1556 (51.2)	<0.01
Female	2339 (50.9)	854 (54.8)	1485 (48.8)	
**Outcome**				
Discharge	4131 (89.8)	1371 (88)	2760 (90.8)	<0.01
Death	468 (10.2)	187 (12)	281 (9.2)	
**Comorbidity**				
Diabetes	1219 (26.5)	708 (45.4)	511 (16.8)	<0.01
Hypertension	593 (12.9)	380 (24.4)	213 (7)	<0.01
BND	221 (4.8)	69 (4.4)	152 (5)	0.39
KD	176 (3.8)	79 (5.1)	97 (3.2)	<0.01
Malignancy	126 (2.7)	24 (1.5)	102 (3.4)	<0.01
RD	162 (3.5)	57 (3.7)	105 (3.5)	0.72
GID	13 (0.3)	2 (0.1)	11 (0.4)	0.15
BD	28 (0.6)	4 (0.3)	24 (0.8)	0.02
LD	34 (34)	11 (0.7)	23 (0.8)	0.85
Pregnancy	42 (0.9)	0 (0)	42 (1.4)	<0.01
No-Comorbidity	1971 (42.9)	0 (0)	1971 (64.8)	<0.01
Comorbidity	1363 (29.6)	539 (34.6)	824 (27.1)	
Comorbidity 1≤	1265 (27.5)	1019 (65.4)	246 (8.1)	

BND: Brain and neurologic disorder; BD: Blood disorder; GID: GI diseases; LD: Liver disease RD: Respiratory disorder; KD: Kidney diseases.

The distribution of patient with CVD and non-CVD among Ct group revealed that the majority of the patients had a Ct between 21 and 30 (Group B) followed by Ct between 31 and 40 (Group C). Also, mortality rate was significantly high in the three groups of Ct value among patients with CVD, with the highest mortality in those with Ct between 10 and 20 (Group A = 19.9%) and lowest in the group with highest Ct (Group C = 9.7%) (*p*≤0.01). The age distribution among Ct value groups were compared, which are as shown in Table 2. Accordingly, statistically significant relationship was observed between increased age and the Ct value in patients without CVD.

**Table 2:** Demographic and comorbidities information of COVID-19 patients with and without CVD based on Cycle threshold value (Ct)

Variable	N [(%)] of Cycle threshold value (Ct) With CVD (1558)				N [(%)] of Cycle threshold value (Ct Without CVD (3041)			
	
	A (n=171)	B (n=758)	C (n=629)	*p*-value	A (n=274)	B (n=1539)	C (n=1228)	*p*-value
**Age**								
1≥	0	2 (0.3)	0	0.81	7 (2.6)	15 (1)	32 (2.6)	<0.01
2–14	0	1 (0.1)	0		15 (5.5)	18 (1.2)	42 (3.4)	
15–24	0	0	1 (0.2)		12 (4.4)	36 (2.3)	34 (2.8)	
25–44	8 (4.7)	43 (5.7)	36 (5.7)		72 (26.3)	454 (29.5)	305 (24.8)	
45–64	62 (36.3)	299 (39.6)	245 (39)		84 (30.7)	633 (41.2)	470 (38.3)	
65≤	101 (59.1)	411 (54.4)	347 (55.2)		84 (30.7)	381 (24.8)	345 (28.1)	
**Sex**								
Male	83 (48.5)	331 (43.7)	290 (46.1)	0.42	133 (48.5)	817 (53.1)	606 (49.3)	0.09
Female	88 (51.5)	427 (56.3)	339 (53.9)		141 (51.5)	722 (46.9)	622 (50.7)	
**Outcome**								
Discharge	137 (80.1)	666 (87.9)	568 (90.3)	<0.01	231 (84.3)	1386 (90.1)	1143 (93.1)	<0.01
Death	34 (19.9)	92 (12.1)	61 (9.7)		43 (15.7)	153 (9.9)	85 (6.9)	
**Comorbidity**								
Diabetes	86 (50.3)	353 (46.6)	269 (42.8)	0.14	54 (19.7)	247 (16)	210 (17.1)	0.30
Hypertension	49 (28.7)	181 (23.9)	150 (23.8)	0.38	25 (9.1)	93 (6)	95 (7.7)	0.07
BND	11 (6.4)	30 (4)	28 (4.5)	0.36	20 (7.3)	76 (4.9)	56 (4.6)	0.16
KD	11 (6.4)	28 (3.7)	40 (6.4)	0.05	18 (6.6)	36 (2.3)	43 (3.5)	<0.01
Malignancy	5 (2.9)	11 (1.5)	8 (1.3)	0.28	13 (4.7)	37 (2.4)	52 (4.2)	0.01
RD	6 (3.5)	25 (3.3)	26 (4.1)	0.70	8 (2.9)	59 (3.8)	38 (3.1)	0.50
GID	0 (0)	1 (0.1)	1 (0.2)	0.87	1 (0.4)	3 (0.2)	7 (0.6)	0.26
BD	0 (0)	1 (0.1)	3 (0.5)	0.35	5 (1.8)	9 (0.6)	10 (0.8)	0.10
LD	0 (0)	5 (0.7)	6 (1)	0.40	4 (1.5)	10 (0.6)	9 (0.7)	0.35
Pregnancy	0 (0)	0 (0)	0 (0)	-	5 (1.8)	23 (1.5)	14 (1.1)	0.58
No-Comorbidity	0 (0)	0 (0)	0 (0)	0.13	160 (58.4)	1035 (67.3)	776 (63.2)	<0.01
Comorbidity	47 (27.5)	267 (35.2)	225 (35.8)		78 (28.5)	391 (25.4)	355 (28.9)	
Comorbidity 1≤	124 (72.5)	491 (64.8)	404 (64.2)		36 (13.1)	113 (7.3)	97 (7.9)	

A: Ct 10–20; B: Ct 21–30; C: Ct 31–40

BND: Brain and neurologic disorder; BD: Blood disorder; GD: GI diseases; LD: Liver disease RD: Respiratory disorder; KD: Kidney diseases

In both groups, there was a significant relationship between outcome of patients age (Table 3). As shown in (Table 3), death patients with history of CVD had a significantly higher rate of kidney disease and hypertension (P-value≤0.01), while, death patients without history of CVD had a significantly higher rate diabetes, hypertension and respiratory disease (P-value≤0.05). The demographic data comorbidities among discharged and death patients are presented in Table 3.

**Table 3:** Demographic and comorbidities information of COVID-19 patients with and without CVD based on outcome.

Variable	With CVD			Without CVD		
	
	Discharged (N=1371) Number (%)	Death (N=187) Number (%)	*P* value	Discharged (N=) Number (%)	Death (N=) Number (%)	*P* value
**Age**						
1≥	2 (0.1)	0 (0)	<0.01	53 (1.9)	1 (0.4)	<0.01
2–14	1 (0.1)	0 (0)		75 (2.7)	0 (0)	
15–24	1 (0.1)	0 (0)		79 (2.9)	3 (1.1)	
25–44	84 (6.1)	3 (1.6)		800 (29)	31 (11)	
45–64	553 (40.4)	53 (28.3)		1091 (39.6)	96 (34.2)	
65≤	728 (53.2)	131 (70.1)		660 (23.9)	150 (53.4)	
**Sex**						
Male	610 (44.5)	94 (50.3)	0.13	1394 (50.5)	162 (57.7)	0.2
Female	761 (55.5)	93 (49.7)		1366 (49.5)	119 (42.3)	
**Comorbidity**						
KD	62 (4.5)	17 (9.1)	<0.01	85 (3.1)	12 (4.3)	0.27
Diabetes	617 (45)	91 (48.7)	0.34	442 (16)	69 (24.6)	<0.01
Hypertension	355 (25.9)	25 (13.4)	<0.01	184 (6.7)	29 (10.3)	0.02
Malignancy	21 (1.5)	3 (1.6)	0.94	89 (3.2)	13 (4.6)	0.21
RD	46 (3.4)	11 (5.9)	0.08	87 (3.2)	18 (6.4)	<0.01
LD	10 (0.7)	1 (0.5)	0.76	20 (0.7)	3 (1.1)	0.52
GID	2 (0.1)	0(0)	0.60	11 (0.4)	0(0)	0.28
BD	3 (0.2)	1 (0.5)	0.42	24 (0.9)	0 (0)	0.11
BND	57 (4.2)	12 (6.4)	0.15	138 (5)	14 (5)	0.99
Pregnancy	0 (0)	0 (0)	-	42 (1.5)	0 (0)	0.37
No-Comorbidity	0 (0)	0 (0)	0.78	1816 (65.8)	155 (55.2)	
Comorbidity 1≤	895 (65.3)	124 (66.3)		733 (26.6)	91 (32.4)	<0.01
Comorbidity	476 (34.7)	63 (33.7)		211 (7.6)	35 (12.5)	

BND: Brain and neurologic disorder; BD: Blood disorder; GID: GI diseases; LD: Liver disease

RD: Respiratory disorder; KD: Kidney diseases.

Of the CVD patients, 54.8% and 45.2% were female and male, respectively. There was no difference in the male and female groups with CVD in terms of different comorbidity. In the non- CVD groups, the most common comorbidity among female patients were diabetes and hypertension and the results revealed a significantly higher prevalence of comorbidities including diabetes (18.7% 15%, p≤0.01) and hypertension (8.3% vs. 5.8%, p≤0.01). Moreover, statistical analysis showed that the presence of at least one comorbidity significantly different among male and female patients in both groups (*p*≤0.01) as shown Table 4.

**Table 4:** Demographic and comorbidities information of COVID-19 patients with and without CVD based on sex

Variable	With CVD			Without CVD		
	
	Female (N=854) Number (%)	Male (N=704) Number (%)	*P* value	Female (N=1485) Number (%)	Male (N=1556) Number (%)	*P* value
**Age**						
1≥	1 (0.1)	1 (0.1)	0.13	22 (1.5)	32 (2.1)	<0.01
2–14	0 (0)	1 (0.1)		31 (2.1)	44 (2.8)	
15–24	0 (0)	1 (0.1)		47 (3.2)	35 (2.3)	
25–44	51 (6)	36 (5.1)		425 (28.6)	406 (26.1)	
45–64	353 (41.4)	253 (35.9)		605 (40.8)	582 (37.4)	
65≤	447 (52.5)	412 (58.5)		354 (23.9)	456 (29.3)	
**Outcome**						
Discharge	761 (89.1)	610 (86.6)	0.13	1366 (92)	1394 (89.6)	0.02
Death	93 (10.9)	94 (13.4)		119 (8)	162 (10.4)	
**Comorbidity**						
Diabetes	428 (50.1)	280 (39.8)	<0.01	278 (18.7)	233 (15)	<0.01
Hypertension	217 (25.4)	163 (23.2)	0.30	123 (8.3)	90 (5.8)	<0.01
BND	44 (5.2)	25 (3.6)	0.12	81 (5.5)	71 (4.6)	0.25
KD	39 (4.6)	40 (5.7)	0.31	41 (2.8)	56 (3.6)	0.18
RD	31 (3.6)	26 (3.7)	0.94	50 (3.4)	55 (3.5)	0.80
Malignancy	13 (1.5)	11 (1.6)	0.94	54 (3.6)	48 (3.1)	0.39
GID	1 (0.1)	1 (0.1)	0.89	6 (0.4)	5 (0.3)	0.70
BD	2 (0.2)	2 (0.3)	0.84	9 (0.6)	15 (1)	0.26
LD	7 (0.8)	4 (0.6)	0.76	12 (0.8)	11 (0.7)	0.74
No-Comorbidity	0	0	<0.01	915 (61.6)	1056 (67.9)	<0.01
Comorbidity	264 (30.9)	275 (39.1)		419 (28.2)	405 (26)	
Comorbidity 1≤	590 (69.1)	429 (60.9)		151 (10.2)	95 (6.1)	

BND: Brain and neurologic disorder; BD: Blood disorder; GD: GI diseases; LD: Liver disease RD: Respiratory disorder; KD: Kidney diseases

## Discussion

Patients with underlying medical disorders are at increased risk for severe illness and enhance vulnerability to COVID-19. CVD is one of the most important underlying diseases which could affect the prognosis of patients with COVID-19 (23, 24). In this retrospective study, we investigated the outcomes and demographic characteristics of COVID-19 patients with and without a history of CVD (25).

This study revealed that CVDs may be important risk factors for severe COVID-19. As shown in Figure 1 and Table 1, there is a significant difference between the age and sex of the COVID-19 patients with and without CVDs. Based on the results of the present study, being older than 45was strongly associated with in-hospital mortality among CVD patients. This is in agreement with Korean study, where found that aging can cause changes in the blood vessels and heart that may increase a person's risk of developing CVD (26). This is also in accordance with another finding of the present study that shows the chance of death increases with age; age demonstrated the highest and closest association with the consequence of the COVID-19;

In Saudi Arabia, a study in 2021 declare that these results may be related to the genetic differences, the lower socio-economic conditions, malnutrition, restricted access to the healthcare facilities (27). In another study, showed that aging is related to decline in various aspects of immune function. Immunosenescence, an augmented inflammation and autoimmunity compresence of a state of immunodeficiency is a novel concept that showed age-related immunological changes (28).

Women's insensitivity to infections can be due to the X chromosome and sex-related hormones, which play a role in the innate and adaptive immunity. However, estrogen (E2) levels decrease with age and menopause status while CVD events increase with age in women (29–31).

Several studies in Iran emphasize that age and sex are associated with severity and death in patients with COVID-19. These studies showed that male gender, older age and existence of at least one comorbidity, especially CVD were significantly associated with the mortality rate among COVID-19 patients (32–34). These researchers suggested that preventive programs to support the elderly and special attention to male elderly cases with underlying disease are essential. Also, it is necessary to focus on the specific health needs of the elderly, such as nutrition, vaccination, continuously check-up, wellbeing, and mental health promotion (35).

In consistence with the results of the present study, a study from Iran showed that COVID-19 cases with a history of CVD such as hypertension and CAD (coronary artery disease), particularly those in specific age and sex groups, are high-risk patients for in-hospital mortality (23).

Interestingly, another report from Iran declared that there was no significant relationship between gender and COVD-19-related mortality, but the survival rate in patients aged > 75 years is reduced to 40% (36). Out of all patients, 42.9% did not have any comorbidity. DM, Hypertension, BND, KD, Malignancy, RD and LD were the most common comorbidities with 26.5%, 12.9%, 4.8%, 3.8%, 2.7%, 3.5% and 34%, respectively. This finding is consistent with two studies from Iran (33) and Korea (26).

In a study in 2020 from Iran showed that case fatality ratio (CFR) in patients with at least one comorbidity including, diabetes, CVD, RD, hypertension, CKD and malignancy was 9.7%, 10.8%, 15%, 13.5%, 16.7% and 5.9%, respectively (33).

The interesting findings of a meta-analysis study show that CVD and its most important risk factors (hypertension and DM) were closely related to CFR in COVID-19 in all age groups. While, young cases had a lower occurrence rates of CVD comorbidities than elderly, relative risk of fatal outcome in young with diabetes, hypertension and CVD was higher (37). Moreover, in a meta-analysis study, report that among comorbidities, hypertension, chronic heart failure, and coronary heart disease were the most commonly described (38). However, in contrast to our results, a cross-sectional study revealed that the proportion of SARS-COV-2 Infection in the Iranian CVD patients was 14.0 % (17).

The present study demonstrated that mortality rate was significantly high among the three groups of Ct value in patients with CVD, with the highest mortality in those with Ct between 10 and 20 (Group A) and lowest in the group with highest Ct (Group C) (p≤0.01). From the point of view of Ct value, lower Ct value are considered to be prognostic factors of death in patients with COVID-19

In a study conducted from Turkey showed that viral burden was not a critical factor for hospitalization and mortality (39). A multivariable logistic regression results showed that there is a significant negative relationship between the odds of death and Ct values (40). In agreement with current funding, a systematic review and meta-analysis study declare that high risk of serious illness and death were seen among patients with CT <25 compared with CT >30 (41).

A retrospective cohort study in the New York city showed that cases with a short period of symptoms and high comorbidity, as well as transplant recipients, were more likely to have a high SARS-CoV-2 genomic burden at the time of hospitalization (42). Regardless of age, comorbidity, severity of disease at the time of admission, cases with high viral burden had more intense clinical manifestations and were twice as likely to die or be intubated. However, in Italian study showed that in comparison with group C patients (Ct ≥ 28.0), the need for hospitalization in group A patients (Ct ≤ 20.0) was higher (56.7% vs. 74.5%, P = 0.031) (43). Severe consequences of COVID-19 were significantly worse in group A than either B (20.0 < Ct ≤ 28.0) or C. Moreover, CFR was higher in group A (36.4%) as compared with B (12.7%) and C (5.6%).

In contrast, a study showed that the SARS-CoV-2 viral burden (inverse relationship with Ct) did not reflect the increasing severity of pulmonary lesions on chest computed tomography (CT). Probably, small sample size (*n=* 158 PCR positive patients) is one of the main reasons for this limitation (44). As an interesting finding in a study, revealed that the low level of the Ct value of SARS-CoV-2 can deteriorate patients' condition and that most of the patients in ICU had low Ct value of SARS-CoV-2. Moreover, their finding showed most of patients with hypertension, and hospitalized patients had low Ct value and more susceptible to shift in ICU (45).

There were some limitations in present study. First, we analyzed only the Ct value of a single time sample, so we could not assess viral load dynamics overtime. Secondly, because laboratory data were not available at the time of the study, we did not incorporate laboratory variables. Another limitation of our work is the inability to check the medical records of CVD patients in order to confidently determine their CVD type.

In sum, current finding highlight that CVD is a major risk factor for hospitalization and the severe consequences of COVID-19. Patients with CVD had significantly more co-morbidities such as hypertension, kidney disease (KD), and diabetes (DM). Also, death in CVD group is significantly higher compared to non-CVD. Moreover, mortality rate was significantly high between the three groups of Ct value among patients with CVD, with the highest mortality in those with Ct between 10 and 20. Furthermore, to improve and validate our findings, well-designed, large-scale, multicenter investigations are required.
